# Supplementary material for: A pan-cancer analysis of homeobox family: expression characteristics and latent significance in prognosis and immune microenvironment
Source: Front Oncol. 2025 Feb 6;15:1521652. doi: 10.3389/fonc.2025.1521652 (PMC11840236; doi:10.3389/fonc.2025.1521652)
Supplement: Supplementary Figure 1 — Mutations of each HOX gene in 32 cancers (TCGA, PanCancer Atlas) by cBioportal. [file DataSheet1.zip › Suppl.files/raw code.zip!/code/4.survival/survivalplot.zip!/survivalplot/HOXD13_OS.THYM.pdf]

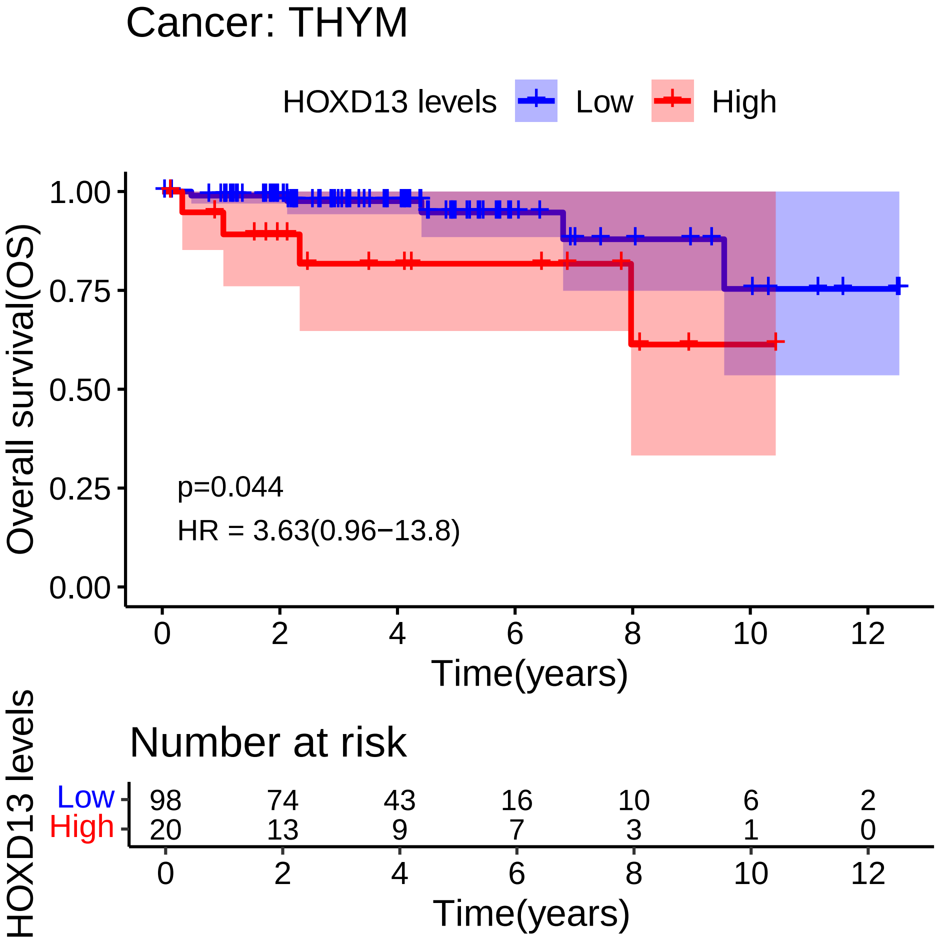

Cancer: THYM
HOXD13 levels
Low
High
Overall survival(OS)
1.00
0.75
0.50
0.25
0.00
p=0.044
HR = 3.63(0.96−13.8)
0
2
4
6
8
10
12
Time(years)
Number at risk
HOXD13 levels
Low
High
98 74 43 16 10 6 2
20 13 9 7 3 1 0
0
2
4
6
8
10
12
Time(years)
